# Nextgen Vector Surveillance Tools: sensitive, specific, cost-effective and epidemiologically relevant

**DOI:** 10.1186/s12936-020-03494-0

**Published:** 2020-11-25

**Authors:** Robert Farlow, Tanya L. Russell, Thomas R. Burkot

**Affiliations:** 1 R Farlow Consulting LLC, Burkeville, TX USA; 2 Australian Institute of Tropical Health and Medicine, James Cook University, Cairns, Australia

**Keywords:** Malaria vector surveillance, Entomological surveillance tools, Laboratory techniques, Next generation tools

## Abstract

**Background:**

Vector surveillance provides critical data for decision-making to ensure that malaria control programmes remain effective and responsive to any threats to a successful control and elimination programme. The quality and quantity of data collected is dependent on the sampling tools and laboratory techniques used which may lack the sensitivity required to collect relevant data for decision-making. Here, 40 vector control experts were interviewed to assess the benefits and limitations of the current vector surveillance tools and techniques. In addition, experts shared ideas on “blue sky” indicators which encompassed ideas for novel methods to monitor presently used indicators, or to measure novel vector behaviours not presently measured. Algorithms for deploying surveillance tools and priorities for understanding vector behaviours are also needed for collecting and interpreting vector data.

**Results:**

The available tools for sampling and analysing vectors are often hampered by high labour and resource requirements (human and supplies) coupled with high outlay and operating costs and variable tool performance across species and geographic regions. The next generation of surveillance tools needs to address the limitations of present tools by being more sensitive, specific and less costly to deploy to enable the collection and use of epidemiologically relevant vector data to facilitate more proactive vector control guidance. Ideas and attributes for Target Product Profiles (TPPs) generated from this analysis provide targets for research and funding to develop next generation tools.

**Conclusions:**

More efficient surveillance tools and a more complete understanding of vector behaviours and populations will provide a basis for more cost effective and successful malaria control. Understanding the vectors’ behaviours will allow interventions to be deployed that target vulnerabilities in vector behaviours and thus enable more effective control. Through defining the strengths and weaknesses of the current vector surveillance methods, a foundation and initial framework was provided to define the TPPs for the next generation of vector surveillance methods. The draft TTPs presented here aim to ensure that the next generation tools and technologies are not encumbered by the limitations of present surveillance methods and can be readily deployed in low resource settings.

## Background

Vector surveillance will increasingly be critical to the success of national malaria control and elimination programmes in designing, planning and monitoring vector interventions as the number of World Health Organization (WHO) recommended vector control strategies increases beyond insecticide treated nets (ITNs), indoor residual spraying (IRS) and larval source management (LSM) [1, 2] The WHO considers surveillance, including vector surveillance, as a core intervention and recommends countries monitor specific malaria vector indicators according to the recommended control strategies implemented (e.g., ITNs, IRS or LSM) [3]. The primary objectives of vector surveillance, as outlined by the WHO, are to characterize receptivity (a function of vector presence and density to enable selection and stratification of interventions), to track malaria vector densities (for selection and timing of vector control deployment by biting time or seasonality of transmission), to monitor insecticide resistance (IR) for selecting insecticides for programme use, to identify other threats to vector control efficacy and to identify gaps in vector control intervention coverage [4]. Monitoring these objectives requires tracking eight specific vector indicators (i.e., vector occurrence, vector density, blood feeding habits, indoor/outdoor biting, indoor/outdoor resting, insecticide resistance phenotypes, sporozoite infections and larval habitats) as well as indicators for monitoring intervention access and use. These eight vector specific indicators are presently monitored with a limited number of mosquito field sampling tools and laboratory analysis techniques, many of which have been in use for decades [5]. The range of entomological surveillance methods presently available may lack the sensitivity required to detect subtle changes in vector behaviours or may not directly measure the vector behaviours that new vector control tools may impact (e.g., repellency, sugar feeding, mating) [6, 7].

A recent analysis of the capacity of NMCPs to conduct vector surveillance programmes was conducted [8]. On average, only 3.8 or 4.7 of the 8 WHO-recommended indicators were monitored in countries controlling or eliminating malaria, respectively. However, the critical indicator of insecticide resistance phenotype was monitored annually by 78% of countries [8]. Across nearly every country surveyed, the vector surveillance programmes were hampered by a lack of capacity and capability. Largely underlying this was a lack of up-to-date strategic plans that prioritize vector surveillance and include frameworks for decision-making and action [9]. Next generation vector surveillance tools have the potential to overcome the lack of capacity by facilitating the collection of mosquitoes with smarter, more efficient methods and tools that require lower resource inputs. Here the objective was to assess the benefits and limitations of the current vector surveillance tools and techniques and to use this as a baseline for developing draft Target Product Profiles (TPPs) that define the framework to guide the development of next generation techniques. Forty vector control experts were interviewed to assess the frequency with which mosquito sampling techniques were used, the indicators that each technique had monitored, and the strengths and weaknesses of these established vector surveillance techniques.

## Methods

In this study, surveillance methods consist for both field sampling tools to collect vectors (i.e. traps) and laboratory techniques to analyse specimens (i.e., for species identification, parasite detection and identification, insecticide resistance phenotyping and age structure determination; see full listing of techniques across Tables 1, 2, 3, 4, 5, 6). To assess the attributes, strengths and weaknesses of current vector surveillance methods and what vector indicators each tool is actually utilized to monitor, 40 key-informant interviews were conducted. The key-informants, vector control experts in Ministries of Health, NMCPs, university researchers, Centers for Disease Control and Prevention/President’s Malaria Initiative and Innovative Vector Control Consortium staff experienced in malaria research and control in Africa, the Americas and Asia, were interviewed between June 2018 and March 2019.

**Table 1:**
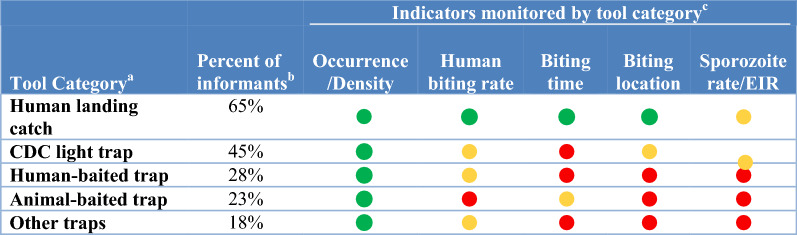
Surveillance tools used to monitor indicators of blood-seeking mosquitoes

^a^The surveillance tools included were: Human landing catch; CDC light trap included CDC light trap with or without lights or lures including placement near an occupied bed net; Human-baited traps included host decoy traps, odour baited entry traps, Ifakara tent traps, the Flavela tent trap, and electrocuting grids near humans; Animal-baited trap included animal baited net traps, animal baited hut traps, Magoon stable traps, and barrier fences around animals; Other fan traps included UV light traps, updraft traps, the BG-Suna trap and the BG-Sentinel trap

^b^The percent of informants regularly using each tool was calculated using the total number of informants (n = 40)

^c^The frequency with which each surveillance category measured each entomological indicator was calculated using the total number of informants using each tool as the denominator. Frequent use (green circle) was defined as more than 50% of informants using the tool to measure the specific indicator; infrequent use (yellow circle) was defined as less than 50% of informants with red circles indicating the tool was not used to measure an indicator by any of the informants

**Table 2:**
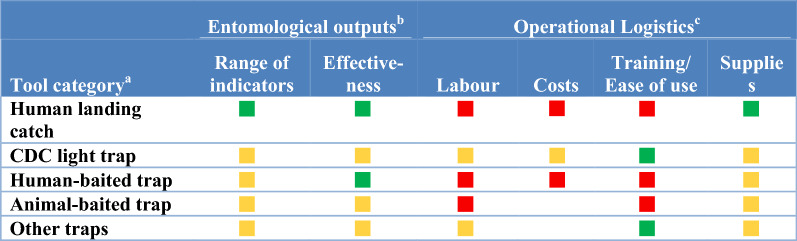
Attributes of surveillance tools targeting blood-seeking mosquitoes

^a^The surveillance tools included in each surveillance category were: human landing catch; CDC light trap (included CDC light traps with or without lights or lures including placement near an occupied bed net); Human-baited traps (included host decoy traps, odour baited entry traps, Ifakara tent traps, the Flavela tent trap), and electrocuting grids near humans; Animal-baited trap (included animal baited net traps, animal baited hut traps, Magoon stable traps, and barrier fences around animals); other fan traps (included UV light traps, updraft traps, the BG-Suna trap and the BG-Sentinel trap)

^b^The entomological outputs assessed each tool as a function of the numbers of entomological indicators monitored by surveillance tool category and effectiveness. For “range of indicators”, a green square indicates the tool monitored almost all indicators in Table 1 while a yellow square indicated the tool was useful for monitoring a moderate number of the indicators in Table 1. A tool’s “effectiveness” was defined as a function of the numbers of specimens and/or species collected with green squares indicating that the tool was efficient in collecting adequate numbers of samples across a range of species while yellow squares indicated the tool collected fewer specimens or did not collect all anthropophagic species

^c^Green squares indicate that expert informants considered this tool to be advantageous in not requiring a lot of human input for establishing or maintaining, was inexpensive, easy to use and required minimal supplies or were easily accessible for “labour”, “cost”, “ease of use” (limited training required) and “supplies”, respectively. Yellow squares indicated either a range of informant opinions as to operational utility. Red squares indicate that the tool had major limitations: high costs, was labor intensive or hard to use

**Table 3:**
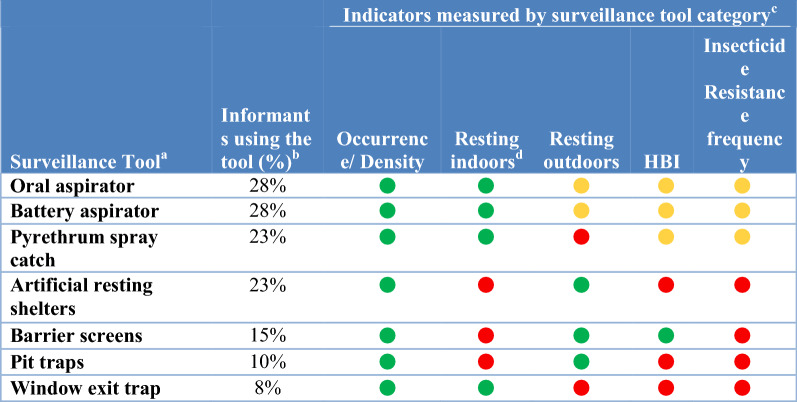
Mosquito surveillance tool attributes for resting and other behaviours

^a^Surveillance tools included oral aspirator (including manual aspirators); battery aspirators (CDC backpack aspirators, bazooka aspirators and Prokopack aspirators); Pyrethrum spray catches (pyrethrum spray and knockdown spray catches using a variety of natural and synthetic insecticides); artificial resting shelters (including clay pot and resting boxes; barrier traps included barrier screens and Malaise traps; pit traps included any iteration of a pit trap); window exit trap included a variety of window exit trap designs

^b^Calculated from a total number of informants of 40

^c^The frequency that each surveillance tool measured each entomological indicator was calculated using the total number of informants that used each tool as the denominator. Frequent use (green circle) was defined as more than 50% of informants used the tool to measure the specific indicator; infrequent use (yellow circle) was less than 50% of informants with red circles indicating the tool was not used to measure an indicator

^d^Indoors includes both inside houses and animal shelters

**Table 4:**
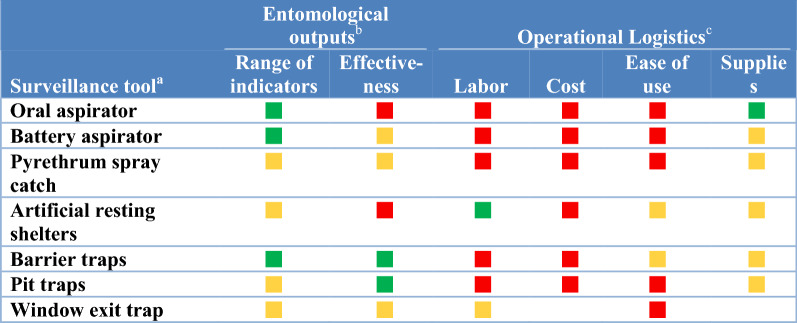
Attributes of surveillance tools targeting resting mosquitoes and other behaviours

^a^Surveillance tools included oral aspirator (includes manual aspirators); battery aspirators (CDC backpack aspirators, bazooka aspirators and Prokopack aspirators); Pyrethrum spray catches (pyrethrum spray and knockdown spray catches using a variety of natural and synthetic insecticides); artificial resting shelters (clay pot, and resting boxes); barrier traps (barrier screens and Malaise traps); pit traps (multiple iterations of a pit trap); window exit trap included a variety of window exit trap designs

^b^The entomological outputs assessed each tool as a function of the numbers of entomological indicators monitored by surveillance tool category and effectiveness. For “range of indicators”, a green square indicates the tool monitored more than one indicator in Table 3 while a yellow square indicated use for monitoring indicators varied by geographic location. A tool’s “effectiveness” was defined as a function of the numbers of specimens and/or species collected with green squares indicating that the tool was efficient in collecting adequate numbers of samples across a range of species while yellow squares indicated the tool collected fewer specimens, did not collect all species or effectiveness varies by location

^c^Green squares indicate that expert informants considered this tool to be advantageous in not requiring a lot of human input for establishing or maintaining, was inexpensive, easy to use and required minimal supplies or were easily accessible for “labour”, “cost”, “ease of use” (limited training required) and “supplies”, respectively. Red squares indicate that the tool had major limitations: high costs, was labour intensive or hard to use. Yellow squares indicated either a range of informant opinions as to operational utility

**Table 5:**
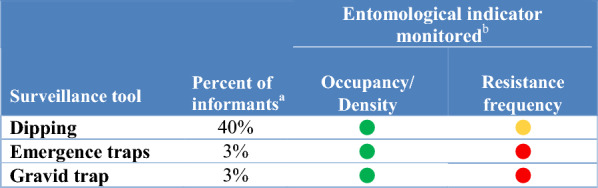
Surveillance tools targeting stages from gravid females through adult emergence

^a^Calculated from the responses from 40 informants

^b^The frequency that each surveillance tool was used to measure each entomological indicator was calculated using the total number of informants that used each tool as the denominator. Frequent use (green)was defined as more than 50% of informants used the tool to measure the specific indicator, infrequent use was less than 50% of informants (yellow) with red indicating the tool was not used to measure an indicator

**Table 6:**
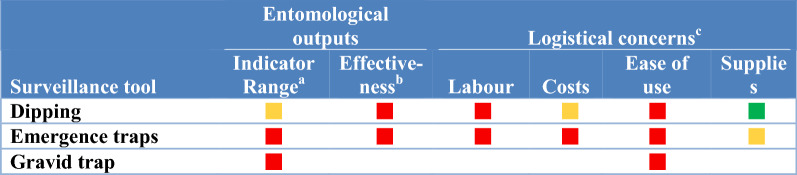
Attributes of surveillance tools targeting immature stages and gravid female mosquitoes

^a^Yellow indicates range of indicators varies by the site, red indicates tool is used for only one indicator in Table 5

^b^Red denotes effective in monitoring only a few species or collects a limited number of species in low numbers

^c^Green squares indicate that expert informants considered this tool to be advantageous in not requiring a lot of human input for establishing or maintaining, was inexpensive, easy to use and required minimal supplies or were easily accessible for “labour”, “cost”, “ease of use” (limited training required) and “supplies”, respectively. Red squares indicate that the tool had major limitations: high costs, was labour intensive or hard to use. Yellow squares indicated either a range of informant opinions as to the scaler of logistical concerns for using the tool

Semi-structured interviews were conducted in person or virtually (see Additional file 1). The purpose of the interview was explained to the expert informants and they were asked to provide their experiences with current vector surveillance methods. Broad categories of strengths and weaknesses by surveillance method were captured for both tools for field specimen collection and techniques for laboratory analyses of field specimens. Positive attributes and limitations of each method with which informants had personal experience were recorded in the informant’s own words and data on how each method is actually used to monitor vectors. Informants were encouraged to provide input on novel indicators for which we presently do not have the capacity to measure. Such “blue sky” indicators encompassed ideas for novel methods to monitor presently used indicators, to measure novel vector behaviours not presently measured, algorithms for deploying surveillance tools and priorities for understanding vector behaviours critical for collecting and interpreting vector data.

Qualitative data were analysed by coding [10] the responses against a standardized framework to assess the utility, strengths and weaknesses of the vector surveillance methods. For surveillance tools, framework included both entomological outputs and operational logistics. The entomological outputs were: (1) range of indicators, being a function of the numbers of entomological indicators monitored by surveillance tool, and (2) effectiveness, being a function of the numbers of specimens and/or species collected by the tool. The operational logistics were: (1) labour, being the requirement for human input for establishing or maintaining the surveillance tool, (2) cost, being a function of both initial outlay and running costs, (3) training/ease of use, being the relatively simplicity to deploy the surveillance tool, and (4) supplies, being the difficulty of replacing consumables. For laboratory analytical techniques, the framework was based on operational logistics including: (1) training requirement, (2) human resource needs, (3) complexity of method, (4) costs/logistics/supplies; (5) specimen quality; (6) in-country capability; (7) interpretation of result; and (8) technical consistency.

## Results

The results that follow summarize the semi-structured interviews and represent the current perceived advantages and disadvantages for various commonly used *Anopheles* surveillance tools and techniques by the global community.

### Sampling adult mosquitoes

The tools used to sample blood-seeking adult mosquitoes were classified into five categories: the human landing catch (HLC); CDC light trap (with or without lights or lures including placement near an occupied bed net) [11]; human-baited traps (including host decoy [12], odour baited entry [13], Ifakara tent [14], Flavela tent [15] and electrocuting grids near humans [16]); animal-baited traps (i.e., animal baited nets [17], animal baited huts, Magoon stable traps [18], and barrier fences around animals); other traps (including UV light, updraft, the BG-Suna [19] and the BG-Sentinel [20]) (Tables 1, 2). Each tool category had unique strengths and weaknesses identified by the 40 informants.

#### Human landing catch

The HLC was the most frequently used technique (Table 1). It was considered to be highly effective and was used to monitor more indicators (determination of peak biting behaviours of indoor and outdoor biting abundance by season and hour) than any of the other methods (Table 2). The HLC was a preferred sampling method that is compatible with analyses for sporozoites and species identification. The HLC was the only technique used by informants that directly estimates the epidemiologically relevant indicator: the exposure of humans to biting mosquitoes. Thus, estimates of the biting rate from the HLC are directly used to calculate the entomological inoculation rate (EIR) by multiplying by the sporozoite rate. This technique requires limited training before implementing and is thus compatible with community recruitment for monitoring all human biting mosquitoes.

The potential exposure of collectors to vector borne diseases limits the use of HLC in some countries [21]. The HLC also requires a high level of supervision to maintain quality and sample size is a function of collector attractiveness and collector efficiency, thus impacting sampling reproducibility (e.g., high variances in catch numbers makes it hard to standardize). Another limitation is the logistics of getting the supervisory team and supplies to study sites.

#### CDC light traps

CDC light traps were the second most frequently used tool for monitoring biting vectors (Table 1). CDC light traps were used to provide data on adult presence/densities both indoors and outdoors. In addition, community householders are easily trained to operate CDC light traps, thus minimizing the need for supervision and the tool is a good measure of tracking anopheline indoor numbers (Table 2). Informants’ opinions varied about the range of indicators monitored by light traps and the effectiveness of light traps.

Identified weaknesses of light traps are logistics in accessing necessary supplies (lures and batteries) and their initial outlay or running costs. The lure often includes carbon dioxide, usually supplied by dry ice which is not universally available. The cost of batteries, and availability, can also significantly limit light trap use. Collection efficacy varies by species and geographic area (e.g., new world mosquito species are generally not well represented in light trap collections). Light traps also catch a lot of non-target insects which damages mosquito specimens while held in the collection bags (mosquitoes are also damaged when drawn through the fan) which reduces the reliability of morphology-based specimen identifications. Community acceptance of CDC-LT indoors is not universal. There is also not a standard operation procedure for the CDC-LT, with deployment varying by light source (visible, UV or none), lure (e.g., CO_2_, near human under bed net or other), lure source (e.g. dry ice, tank gas) and sampling site location (inside and outside of houses) making comparisons uncertain across geographic locations.

Data from CDC-LT are not directly epidemiologically relevant (i.e., the number of mosquitoes collected must be transformed to a biting rate which will vary by species, human biting habit and geographic area and requires simultaneous catch comparisons in any new area to HLC to validate interpretation of catch abundance. When used outdoors, the interpretation of the number sampled is uncertain (i.e., the range that mosquitoes are attracted to the CDC-LT is unknown).

#### Human and animal baited traps

Human and animal-baited traps were used less frequently in studies of biting mosquitoes and were limited to providing data on biting rates and times (Table 1). Human-baited traps were considered to be effective, while animal baited traps were reported to have variable effectiveness.

Both human and animal baited traps were limited by labour, costs and ease of use. The relationship of the number of sporozoite positive mosquitoes captured when attracted to animal baited traps to the sporozoite rate in mosquitoes attracted to humans was uncertain to informants and thus concerning, as was the relative attractiveness of mosquitoes to animals and humans; thereby making the calculation of the EIR based on these techniques problematic without area specific comparisons to HLC collections.

### Resting mosquitoes

Two basic surveillance categories sampled resting mosquitoes: sampling natural resting habitats with aspirators and knockdown spray catches and the construction of sites from which resting mosquitoes are collected (e.g., barrier screens [22], pit traps and window traps) (Tables 3, 4).

#### Sampling natural habitats

Searches for resting mosquitoes with oral and battery-powered aspirators or collections following knockdown (i.e. pyrethrum) sprays were the most common tools for sampling indoor resting mosquitoes. None of these tools were considered to be particularly effective (Table 4). These three tools generate data on indoor density and for determining the human blood index and to provide samples for analyses for insecticide resistance frequency. However, these tools are labour intensive (and thus costly) and logistically not easy to implement (Table 2).

#### Sampling man-made habitats

The second approach is to create or provide suitable resting habitats prior to collection through building of pit traps, provision of resting boxes and clay pots [23] and construction of barrier screens [24]. Pit traps often catch a lot of mosquito specimens (Table 4). However, the technique is labour intensive to set up and to maintain. Resting pots/boxes are relatively immobile and capture few mosquitoes.

Barrier screens differ from both pit traps and resting boxes and pots in that the barrier screen intercepts mosquitoes in transit to likely resting habitats or when transitioning between behaviours (e.g., while seeking oviposition sites, blood or sugar meals or mating swarms). Barrier screens are inexpensive to construct from locally sourced materials, and community members are easily trained to construct and operate barrier screens. Barrier screens are labour intensive to operate and involve some effort to transport and assemble materials in remote sites.

There was not a preferred tool for sampling resting adults. The barrier screen, the most recently developed tool, measured more indicators associated with resting mosquitoes but was used by only 15% of informants. The need for a new more efficient and less labour-intensive tool for sampling resting mosquitoes was universally expressed.

### Sampling immatures and gravid adults

Dipping [25] and emergence traps [26] were used to monitor immature populations and gravid traps for sampling ovipositing adults [27] (Tables 5, 6). Dipping was the most commonly used tool and uses simple and inexpensive equipment but the effectiveness of dipping varies by site (Table 6), being effective in small larval habitats [though some habitats may be inaccessible or cryptic (i.e., hard to find)]. Moreover, samples from larger larval habitats are biased as only the perimeter is sampled and thus the distribution of larvae is not defined. Larval surveys by dipping are labour intensive (with efficiency of collections varying by individual collectors) and dipping procedures are not standardized.

While dipping for larvae can determine species presence, a significant limitation includes the uncertain relationship of larval numbers to epidemiologically relevant indicators as the density or prevalence of dips positive for larvae to larvae numbers by surface area, total larvae numbers in the habitat or the number of biting adults is unknown. A confounding factor for interpretation of results is that the consistency of dipping by species will vary by species-specific larval behaviours and the efficiency of the collector.

Emergence and gravid traps provide data on mosquito species presence and density but were not used by vector control experts as they do not provide much information on other entomological indicators. These sampling tools are not easy to use and require training before use.

### Analysis of mosquito specimens

Adult mosquito samples were most often analysed for species identification, malaria parasite species infection and adult mosquito age structure. While all the analysis techniques are potentially compatible with in-country analyses, almost all of the techniques require well trained staff, have complex protocols, have significant costs and associated logistic constraints, as well as requiring specimens in good condition. The interpretation of results from these laboratory techniques are susceptible to variability, which can arise from the variation in the technical consistency of the laboratorian performing the test (Table 7).

**Table 7:**
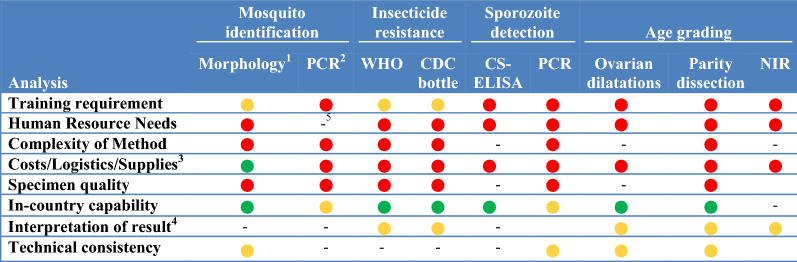
Summary assessment of laboratory analytical techniques for malaria vectors by expert informants

^a^Yellow indicates a moderate level of training required

^b^Red indicates significant requirements for use including high level of training, human resources, complex methodology, costs, need for quality specimens, which impacts technique uptake and use

^c^Green indicates few impediments (few logistics concerns, low costs or in country capability present) for use

^d^Yellow indicates variability in interpretation of results and technical consistency

^e^“-”, not expressly addressed by informants

### Mosquito identification

Thirty-eight percent of vector control experts commented on vector identification techniques.

#### Morphological mosquito identification

Morphological mosquito identification is less expensive than molecular-based identifications, and countries have this capability. However, the training and retention of staff to use complex taxonomic keys are drawbacks. Morphological-based identifications are often only to species complexes even for good condition specimens or a high identification error rate can result (varies by site/person). Hence, morphological identifications almost always require molecular confirmation for members of species complexes. Ethanol storage for ease of molecular identifications, unfortunately, increases the difficulty of morphological identifications.

#### Molecular mosquito identifications

Polymerase chain reactions (PCR) for mosquito identifications can be done in some countries and PCR based analyses is compatible with multiple storage conditions and has high sample throughput [28]. However, the technique requires a high level of training and the protocol is complex with high costs. A prerequisite for PCR-based analyses is accurate morphological identifications prior to molecular analyses which requires high quality specimens. While PCR reactions can be done in-country, some countries send specimens outside the country for sequencing, (Table 7).

### Parasite detection in mosquitoes

Ten vector control experts critiqued the circumsporozoite enzyme-linked immunosorbent assay (CS-ELISA) and PCR to identify malaria parasites in mosquitoes. Both techniques share the need for a high level of training, have high laboratory establishment costs, are labour intensive techniques with complex analyses protocols. However, both techniques are compatible with multiple mosquito storage methods prior to analyses.

#### CS-ELISA

The CS-ELISA strengths are species specificity, lower laboratory establishment and sample run costs relative to PCR and compatibility with analyses of pools of mosquitoes [29]. Once established, the CS-ELISA was judged to be more robust and reliable than PCR by the key informants (Table 7). However, the need for PCR analysis for mosquito species identifications is a significant limitation as CS-ELISA analyses does not negate the expense of conducting PCR based assays to identify the vector species in species complexes.

#### PCR sporozoite detection

PCR can identify sporozoites to species [30] and is compatible with PCRs for mosquito species identifications and insecticide resistance mechanism determination, a significant advantage over the CS-ELISA.

The PCR for sporozoite detection is more costly than the CS-ELISA to process samples and to establish the laboratory. The technique is species but not stage specific, so, like the CS-ELISA, mosquito heads and thoraxes need to be separated from abdomens before analysis. There is also the potential for interference by blood contamination if abdomens are not removed prior to analyses.

### Age grading

The current techniques to age grade field collected mosquitoes are dissections for parity and ovarian dilatation counts [31]. A third technique, near infra-red scanning, has not yet been evaluated on wild mosquitoes [32]. Seventeen vector control experts provided input on these three-mosquito age-grading techniques.

#### Ovarian dilatations

The advantage of ovarian dilatations for age grading mosquitoes is the sensitivity of age determinations based on the number of oviposition events which provides epidemiology relevant data (i.e., the potential to differentiate mosquitoes that have lived long enough to transmit pathogens from younger mosquitoes) (Table 7). Ovarian dilatation dissections are labour intensive and challenging. Significant training/practice is required, and variability in scoring the number of dilatations can be high. A microscope is required.

#### Parity dissection

Parity dissections are easier to perform than ovarian dilatation dissections and parity determination is thus a field friendly technique, enabling a skilled technician to age-grade many mosquitoes by distinguishing parous from non-parous mosquitoes. Weaknesses of parity dissections were the lack of specificity (mosquitoes either have never laid eggs or they have laid eggs), a microscope is required, and live mosquitoes must be dissected (limitations shared with the ovarian dilatation technique). While easier than ovarian dilations, experience and practice are required; the technique is labour intensive and needs a dedicated team.

#### Near infra-red

The relatively new technique of using near infra-red scanning to age-grade mosquitoes has advantages of being compatible with high throughput, is inexpensive to analyse mosquitoes (after the initial establishment costs) and is compatible with species identification [33]. However, there is significant variance in the age estimations of laboratory specimens of known age. Specimens must be carefully handled and oriented prior to scanning. Establishing the age of a mosquito requires calibration against mosquitoes of known chronological age from each area and time when the technique is used. The calibration curve is labour intensive to establish and its stability in time and space is unknown. Thus far, the reliability and usefulness of near infra-red analyses for determining mosquito population age-structures has not been verified by studies of wild mosquito populations.

#### Insecticide resistance and quality assurance

Current techniques for measuring insecticide resistance phenotypes in mosquitoes were discussed by 15 vector control experts. Training requirements, protocol complexity, logistics including transporting specimens to laboratories and the need to rear larvae from field collections to adult mosquitoes were key weaknesses for both the WHO tube test and the CDC bottle bioassay. Updated and more detailed SOPs are needed. Instructional videos would help in consistently defining live and dead mosquitoes.

#### WHO tube test

The WHO tube test is the historical gold standard and is compatible with both laboratory and field-based analyses [34]. The existence of extensive historic data enables comparisons and tracking of resistance trends. However, WHO tube test analyses are limited to fast acting contact insecticides for whom the resistance thresholds are poorly defined. The stock supply of WHO insecticide-treated papers, particularly papers with different concentrations of an insecticide for intensity assays, varies with papers sometimes arriving with a limited use window before exceeding the expiry dates (Table 7). Furthermore, it is uncertain if the discriminating dose as applied to the papers are appropriate for all species. The construction of the tubes and slides is poor, making manipulation of mosquitoes difficult and the duration of mosquito contact with the insecticide paper is uncertain.

#### CDC bottle bioassay

The CDC bottle bioassay measures shifts in population insecticide resistance phenotypes and is more adaptable to local conditions than the WHO tube test. The available SOPs lack sufficient detail and would benefit from videos on defining live and dead mosquitoes. Assays using the PBO synergist are challenging in that they require a 1-h pre-exposure to PBO before exposing mosquitoes to pyrethroids, which damages mosquitoes and thus impacts survival. Errors are easily made in the dilution series and acetone procurement is difficult in some countries.

#### Bioassay for insecticide concentrations on treated surfaces

The current technique to determine biologically active insecticide concentrations on treated surfaces is the WHO cone test attached to a wall or ITN [35]. Weaknesses identified by 5 informants were inconsistent results by mosquito strains (i.e., lack of a standard mosquito reference strain), which is compounded by lack of a standard mosquito rearing technique (Table 7). The need for colony mosquitoes in assays carries the high operational cost associated with maintenance of an insectary. The cone bioassay technique requires a high degree of training and is labour intensive. Use of WHO cones on uneven mud walls often results in mosquitoes escaping (as maintaining an adequate seal with the wall surface is difficult). As with the WHO tube test, the actual contact time of mosquitoes on the treated surface is unknown.

### Next generation entomological surveillance tools

Twenty-three vector control experts provided ideas and attributes for Target Product Profiles (TPPs) for seven next generation vector surveillance tools based upon the identified strengths and limitations of current surveillance tools, as summarized above. Overall, it was expressed that the next generation of surveillance field tools and surveillance techniques should be simple to deploy with minimal training and manpower and have low establishment and operational costs to collect and process specimens. Sampling tools should be applicable for both indoor and outdoor applications and be amenable to a standardized deployment strategy. New tools should sample populations using representative sampling algorithms to measure epidemiologically relevant parameters that can be quickly and easily interpreted by programme managers for decision-making (see Additional file 2).

Seven vector sampling methods were identified as priority targets for improving vector surveillance in two broad categories: vector sampling (i.e., HLC alternatives, automated adult traps, quantitative larval sampling, vector age-grading and identifying malaria parasites) and insecticide monitoring (i.e., quantitative non-bioassay methods for surface active compounds and insecticide resistance phenotyping).

#### Vector sampling

*HLC alternatives* Alternatives to the HLC are needed to sample human host-seeking adult vectors that can be calibrated to historical HLC data without placing trap operators at risk and are safe for use in or near human dwellings. Any new method must maintain the functionality of the HLC (see Table 1) and monitor without bias the presence of all malaria vector species in an area, regardless of their density or behaviours (time of biting, anthropophagy, resting habits) (see Additional file 2). Alternatives to the HLC could include automated sensitive and specific traps to attract, collect, count and identify mosquitoes to species to improve data collection and specimen quality. Like any HLC alternative, automated trap catches should be compatible with calibration to current trapping techniques but capable of collecting all malaria vector species both indoors and outdoors while recording the number of adult female vectors collected per unit time (see Additional file 2).

*Quantitative larval sampling* A quantitative larval sampling tool to estimate adult malaria vector populations is needed to determine mosquito larval vector composition and densities. Larval sampling methods must fully characterize larval habitats to identify productive sites and to predict habitat creation following rainfall. Autonomous sampling would be beneficial and could be based on drone or other technologies that could sample larvae and identify species with multi-imaging capacities to find water bodies based on species specific characteristics across ecosystems (see Additional file 2).

*Vector age*-*grading* A technique to determine mosquito age in 1-day increments is required to calculate median mosquito age with a 95% confidence interval (see Additional file 2).

*Identifying malaria parasites* A novel rapid diagnostic test to identify all *Plasmodium* species in vector species that can be calibrated to current *Plasmodium* detection and identification techniques is required to reliably identify sporozoites in mosquitoes at a sensitivity of < 1000 sporozoites with a 95% precision in duplicate readings of mosquito cohorts (see Additional file 2).

#### Insecticide monitoring

*Quantitative non*-*bioassay methods for surface*-*active compounds* A technique to quantify surface-accessible active compounds as a cost-effective alternative to WHO cone bioassays or chemical extraction and analysis techniques is needed to circumvent the cost of maintaining mosquito colonies. The technique needs to be field applicable, rapid and accurate in detecting all active ingredients and chemical stereoisomer ratios and content on all surfaces and sufficiently robust to operate in the presence of common wall contaminates (e.g. dirt or smoke) with operational costs below current costs to determine surface insecticide concentrations (see Additional file 2).

*Insecticide resistance phenotyping* A cost-effective method to define insecticide resistance phenotypes (frequency and intensity) as well as molecular or biochemical resistance markers in adult mosquitoes that reduces human and budget resources over current WHO tube and CDC bottle assays is needed (see Additional file 2).

#### Research required for effective vector surveillances

Vector control experts noted that little is known about some mosquito behaviours that are being targeted by novel control measures (e.g., outdoor resting and blood feeding, sugar feeding, mating). Thus, there is a need to develop next generation vector surveillance tools to monitor these behaviours. The resulting data will then better inform interventions targeting these behaviours. The following priority areas were identified as critical for improving vector surveillance.

#### Vector behaviour data to inform surveillance deployment

More information is needed on the movement of adult vectors from emergence to blood feeding and oviposition sites to inform representative sampling over a geographic area and not biased to areas with high densities. Such representative sampling can then be used to optimize intervention deployment more effectively. Improved surveillance would benefit from understanding (1) where and when mosquitoes are exposed to control tools, (2) the distribution of daytime biting mosquitoes; (3) host attractancy/biting rates of mosquitoes to different blood meal hosts, (4) novel attractants to replace carbon dioxide, human odours and other blood seeking lures to improve trap performance, (5) receptivity (its definition and measurement), (6) outdoor resting site characteristics by species to improve interventions targeting exophilic adults, (7) larval habitat characteristics by species to improve larval source management. Detailed understanding of the biology of vectors will improve both vector surveillance and malaria control programmes.

#### Data management

Faster data entry systems with uniform formats for data capture and recording across locations will enable vector surveillance data to guide programme decisions in real time to improve vector surveillance.

#### Algorithms for representative sampling

Historically the need to maximize mosquito collection samples sizes (e.g., determination of sporozoite rates, age structure and peak biting time) encouraged sampling bias to collect maximum numbers of mosquitoes with minimal effort. Algorithms for representative sampling across geographic areas for adults and larvae as well as defining/stratifying receptivity are essential.

Representative sampling methods for resistance testing of mosquito populations are needed. More spatially explicit representative sampling for resistance phenotypes are required along with guidance on interpreting results for resistance management (i.e., when to increase insecticide concentrations or to switch insecticide classes in the face of changing resistance profiles). Algorithms are needed to correlate vector insecticide resistance bioassay and genetic data to intervention impact and to correlate hut data to intervention efficacy at scale.

## Discussion

Surveillance encompasses the field sampling tools, their deployment strategies and the laboratory techniques to analyse captured vectors. Vector surveillance provides critical data for decision-making to ensure that malaria control programmes remain effective and responsive to threats such as insecticide resistance, behavioural resistance, changes in species composition and invasive species [2, 6, 7, 36–39]. However, a recent global assessment of vector surveillance activities by NMCPs revealed that routine surveillance of key indicators is limited and that entomological data is rarely used to inform programmatic decisions [8]. This suggests that programmes must react to losses in programme effectiveness rather than being proactive in responding to potential threats as they arise, such as behavioural and physiological resistance to insecticides. Underscoring this is a lack of capacity and capability of NMCP to implement vector surveillance, noting that 92% of country programmes identified limitations (out of 35 countries) [9].

The assessment presented here summarizes how global vector control experts have been using the currently available vector surveillance tools and techniques to evaluate new vector control strategies and/or to monitor malaria control programmes. Overall, this analysis identified several consistent strengths and weaknesses shared by both the field tools and laboratory analysis techniques that constrain the scope and scale of vector surveillance undertaken by NMCPs. These weaknesses included high labour and resource requirements (human and supplies) coupled with high outlay and operating costs and variable tool performance across species and geographic regions. The strength of this assessment is that it was based on 40 semi-structured interviews with global control experts and the interviews were coded against a standardized framework of indicators that defined the utility, strengths and weaknesses of the tools. This was a fundamentally different methodological process to previous landscape analyses and as such the results summarize how the community utilizes current methods as opposed to presenting a summary of the potential applications of surveillance methods [40].

Defining the strengths and weaknesses of the current vector surveillance methods provided a foundation to define draft TPPs for the next generation of vector surveillance tools; ones that will not be encumbered by the limitations of our present surveillance methods. Any new tools and techniques will need to be assessed against a standardized framework to ensure non-inferiority relative to present tools as regards both the logistics of deployment and performance (sensitivity and specificity) in a manner analogous to evaluations of new vector control products [41]. Vector surveillance has also been constrained by a number of non-technical factors including a lack of complete understanding of the biology of the vectors, bias in how samples are collected and that vector data is often not epidemiologically relevant but requires translation/interpretation before it is useful. Hence, this manuscript identified as priorities the need for supporting studies to understand vector behaviours and the need for algorithms for representative sampling to measure indicators that can directly predict potential impacts on malaria transmission.

More efficient sampling methods which are less dependent on human resources and less expensive will facilitate increased surveillance (both frequency of sampling and number of sampling locations). Vector surveillance is fundamental to deploying more effective vector control [42], in particular targeting vectors outdoors [6], which requires an understanding of mosquito behaviours, particularly outdoor movements, resting behaviours and larval habitats [43]. Surveillance data needs to be epidemiologically relevant to be used directly in models to guide programmatic choices on interventions and combinations of interventions deployed. This requires concurrent algorithms to translate field vector surveillance data to vector population estimates so that changes in vector data will estimate changes in disease transmission risk. A priority is to develop algorithms which correlate insecticide resistance bioassays with both genetic data and intervention impacts on transmission. Also, important to highlight, is that the key vector indicators to be monitored will change as transmission intensity diminishes and the range of available recommended interventions increases. While sporozoite rates are a recommended key indicator in high transmission scenarios, the challenge of measuring sporozoite and entomological inoculation rates with sufficient precision to guide programmatic decisions becomes more difficult as transmission is reduced to low levels which requires the analyses of increasingly larger numbers of mosquitoes to find those infected with sporozoites. Thus the need to measure biting rates becoming an increasingly important indicator for predicting transmission risk in low transmission areas [44].

Prioritization and investments in new surveillance tool development should be shared by the entire malaria community including but not limited to NGOs, government agencies, research institutes and commercial companies. The challenges and risks associated with the successful development and implementation of new tools are acceptance and recognition for the use of alternative surveillance technique by global health authorities and NMCPs, and (2) the potential that new surveillance techniques may not have broad spectrum effectiveness across disease vectors or ecosystems which may limit the usefulness globally.

## Conclusion

More efficient surveillance tools and a more complete understanding of vector behaviours and populations will provide a basis for more cost effective and successful malaria control by better enabling interventions to be selected that align with vulnerabilities in vector behaviours and thus enable more effective control. Vector surveillance methods, as has been said for vector control interventions, are imperfect tools applied imperfectly, but present vector surveillance methods have been useful in assessing the potential of new control methods and for guiding the implementation of recommended malaria vector control strategies. The draft TTPs presented here aim to ensure that the next generation tools and technologies are not encumbered by the limitations of present surveillance methods and can be readily deployed in low resource settings. Improvements in the quality, quantity and availability of entomological data will no doubt facilitate more proactive vector control guidance and support the global progress towards malaria eradication.

## Supplementary information

**Additional file 1.** Semi-structured interview guide.

**Additional file 2.** Nextgen vector surveillance draft TPPs.

## Data Availability

Summarized responses to the semi-structured interviews are available upon request.

## Abbreviations

CDC: Centers for Disease Control and Prevention
IRS: Indoor residual spraying
ITN: Insecticide-treated nets
IVCC: Innovative Vector Control Consortium
IVM: Integrated vector management
LLIN: Long-lasting insecticide-treated net
LSM: Larval source management
NMCP: National Malaria Control Programme
PMI: President’s Malaria Initiative
WHO: World Health Organization
