# Supplementary material for: Nextgen Vector Surveillance Tools: sensitive, specific, cost-effective and epidemiologically relevant
Source: Malar J. 2020 Nov 25;19:432. doi: 10.1186/s12936-020-03494-0 (PMC7687713; doi:10.1186/s12936-020-03494-0)
Supplement: Supplementary file 1 — Additional file 1. Semi-structured interview guide. [file 12936_2020_3494_MOESM1_ESM.docx]

**Additional File 1. Semi-structured interview guide**

**Objective:** The objective is to develop target product profiles for the next generation of vector surveillance tools. The process is to first describe how well the present surveillance tools we use work (what they do well as well as their limitations). We will then use these descriptions to create target product profiles that describe a better way of measuring important vector characteristics including “blue sky” tools to measure parameters of interest for which we presently have no means of measuring.

**Improving Vector Surveillance tools**: Please consider the tools that you have used (a partial listing of possible tools follows and consider the advantage and disadvantages (what they do well, what you wish they could do better). This might include things like the following:

cost, training required, established gold standard, ease of comparison across sites, level

of standardization (protocols), ease of interpretation of results, field applicability, throughput, sensitivity, specificity, community acceptance, reliability, manpower requirements, etc.

**Blue Sky Techniques**? Are there any parameters that you wish we could measure but do not have the means to do so now? Let me know and we can communicate to the Gates Foundation to encourage investments to develop new techniques.

**Vector Surveillance tools in use**

Adult mosquito density tools

Human Landing catches

Traps

CDC light trap

CDC light trap near bednet

UV light trap

Sunia trap

Resting collections

Aspiration of resting indoor

Aspiration of resting outdoors

Barrier screens

Prokopec aspirator collection

Pyrethrum knockdown stray catch

Resting boxes, pots

Pit shelters

Mosquito Identification

Molecular identification

Morphological Identification

Parasite detection in mosquitoes

CS-ELISA

PCR detection

Larval surveys

Finding larval habitats

Dipping for larvae

Survivorship estimation

Parity dissection

Ovarian dilatations

Near Infra-Red

Insecticide resistance

CDC bottle bioassay

WHO tube test

Insecticide resistance mechanism

Insecticide availability

Cone tests

**Surveillance tool interview questions**

Name and Institutional Affiliation:

Intervention type (e.g. LLIN, ATSB):

| **Surveillance tool 1** (landing catch, light trap, parity dissection, CS ELISA, PCR for identification) | Name |
| --- | --- |
| **End points measured** (e.g, adult density, time of biting, survivorship, sporozoite rate, insecticide resistance, mosquito identification) |  |
| **Advantages** (e.g., low cost, minimal training, gold standard, high sensitivity, specific, ease of standardization, community acceptance, etc.) | **Limitations** of this surveillance tool (high cost, technical difficulty, unreliable, ethical concerns, non-specific, etc.) |
|  |  |
|  |  |
|  |  |
|  |  |
|  |  |
| **Surveillance tool 2** (landing catch, light trap, parity dissection, CS ELISA, PCR for identification) |  |
| **End points measured** (e.g, adult density, time of biting, survivorship, sporozoite rate, insecticide resistance, mosquito identification) |  |
| **Advantages** (e.g., low cost, minimal training, gold standard, high sensitivity, specific, etc.) | **Limitations** of this surveillance tool (high cost, technical difficulty, unreliable, ethical concerns, non-specific, etc.) |
|  |  |
|  |  |
|  |  |
|  |  |
|  |  |
| **Surveillance tool 3** (landing catch, light trap, parity dissection, CS ELISA, PCR for identification) |  |
| End points measured (e.g, adult density, time of biting, survivorship, sporozoite rate, insecticide resistance, mosquito identification) |  |
| **Advantages** (e.g., low cost, minimal training, gold standard, high sensitivity, specific, etc.) | **Limitations** of this surveillance tool (high cost, technical difficulty, unreliable, ethical concerns, non-specific, etc.) |
|  |  |
|  |  |
|  |  |
|  |  |
|  |  |

Please add additional rows for more surveillance tools

What are Gaps in current surveillance tools?

What are potential alternative current Surveillance tools that might fill gaps?

As we approach elimination, are changes in surveillance techniques needed?

What additional information/data regarding vectors and vector control is needed?

Blue sky

For future Surveillance tools to fill the gaps would you like to see developed?

What end points to be measured?

Describe attributes of new tool.

|  |  |  |  |  |  |  |  |  |  |  |  |  |  |  |  |  |  |  |
| --- | --- | --- | --- | --- | --- | --- | --- | --- | --- | --- | --- | --- | --- | --- | --- | --- | --- | --- |
