# Supplementary material for: Nextgen Vector Surveillance Tools: sensitive, specific, cost-effective and epidemiologically relevant
Source: Malar J. 2020 Nov 25;19:432. doi: 10.1186/s12936-020-03494-0 (PMC7687713; doi:10.1186/s12936-020-03494-0)
Supplement: Supplementary file 2 — Additional file 2. Nextgen vector surveillance draft TPPs. [file 12936_2020_3494_MOESM2_ESM.docx]

**Additional File 2. Nextgen vector surveillance draft TPPs**

**This additional file is part of paper: Farlow R, Russell TL, Burkot TR. Nextgen Vector Surveillance Tools: sensitive, specific, cost-effective and epidemiologically relevant.**

**DRAFT TPP criteria for alternative surveillance technique to human landing catch to determine adult vector composition and behaviour**

- Goal: Develop surveillance techniques to capture human host seeking female mosquitoes
  - Can be calibrated to historical HLC data
- Application method:
  - TBD; Conducted both indoor and outdoor
- Expected performance:
  - Determine the presence of all malaria vector species; (equally effective for collection of adults of all vectors in an area)
  - Determine the number of adult female vectors collected, per unit time
  - Determine adult vector behavior such as, time and location of biting indoors and outdoors
  - Data digitally recorded on site
- User Acceptability
  - Suitable for routine use
- Safety
  - Collection device safe for use in or near Human dwellings
- Target price
  - Reduces human and budget resources to monitor adult Anopheles spp composition and behavior

**DRAFT TPP criteria for novel automated mosquito trap to collect, count and identify to species**

- Goal: Develop novel automated trap to collect, count mosquitoes and identify to species
- Entomological surveillance techniques to capture host seeking female mosquitoes
  - Can be calibrated to current trapping techniques and species identification techniques including PCR
- Application method:
  - TBD; Conducted both indoor and outdoor
- Expected performance:
  - Determine the presence of all malaria vector species; (equally effective for collection of adults of all vectors in an area)
  - Determine the number of adult female vectors collected, per unit time
  - Determine adult vector behavior such as, time and location of biting indoors and outdoors
  - Data digitally recorded on site
  - Results accuracy consistent with at least 98% repeatability in duplicate reading of this same mosquito cohorts at same specific site
- User Acceptability
  - Suitable for routine use
  - Solar powered or other local sustainable power, avoid use of batteries
  - Automated activation
  - Portable and executable at field level
- Safety
  - Collection device safe for use in or near Human dwellings
- Target price
  - Reduces human and budget resources to monitor adult Anopheles spp composition and behavior
  - Reduce labor and resources reduced by at least 20% below current HLC and species identification costs

**DRAFT TPP for quantitative larval sampling to estimate adult vector populations.**

- Goal: Develop novel quantitative larval sampling surveillance technique to detect and estimate malaria vector populations.
  - Cost effective alternative for dipping
- Application method:
  - Conducted in field
  - Suitable for village scale
- Expected performance:
  - Determine mosquito larval vector composition
    - Presence of Anopheles species known to support the development of Plasmodium sporozoites. Requires correct identification of species. Sampling method is not biased for how they swim (think funestus swim differently very hard to catch
  - Capable of sampling wells and overhead water storage sites
  - Determine density of key species
    - The ultimate density of primary regional Anopheles vectors capable of disease transmission. Will need to be calculated to estimate number per unit area, e.g. Sq. km) and expected adult numbers
  - Fully characterize larval water bodies for all malaria vector species
  - Data digitally recorded with GPS coordinates
  - Autonomous sampling
    - Floating drone which can sample larvae and Identify to spp
    - Machine learning by drone to find water in landscape
    - Multi-imaging capacities to find water bodies based on species specific characteristics across ecosystems
- User Acceptability
  - Suitable for routine use
  - Minimal training required
- Safety
  - Safe for use in or near Human dwellings
- Target price
  - Reduces human and budget resources to monitor larval Anopheles spp composition and density

**DRAFT TPP to age grade key malaria mosquito species**

- Goal: Develop novel technique to age grade all malaria mosquito species
  - Can be calibrated to current age grading techniques
  - Cost effective alternative to parity dissection
- Application method:
  - Conducted in field and lab
- Expected performance:
  - Determine mosquito age in 1-day increments Determine mosquito larval vector composition
    - Determine age <5, 5-10 and >10 days old increments as minimum
  - Calculate median age with 95% CI
  - Determine age structure by chronological age, fast and dependable
  - Determine when 90% key vectors are too young to transmit malaria determine when 90% key vectors are too young to transmit malaria
- User Acceptability
  - Suitable for routine use
  - Results obtained on site on less than 5 minutes
  - Minimal training required
  - Low upfront costs
- Safety
  - Safe for use in or near Human dwellings
- Target price
  - Reduces human and budget resources to monitor adult Anopheles spp. age structure
  - Reduce labor and resources below 20% of current Parity dissection

**DRAFT TPP criteria for a Rapid Diagnostic Test to identify Plasmodium in malaria vector species.**

- Goal: Develop a Rapid Diagnostic Test to identify Plasmodium in malaria vector species
  - Can be calibrated to current Plasmodium detection and identification techniques including PCR
  - Cost effective alternative to current Plasmodium detection and identification methods in adult Anopheles
- Application method:
  - Conducted in field and lab
- Expected performance:
  - Develop RDT technique for sporozoite in mosquitoes for all Plasmodium spp
  - Detection level TBD - Target <1000 sporozoites
    - Need lower detection limit in elimination setting
  - Low level of false positives and false negatives
  - Results consistent with 95% precision in duplicate reading of same cohort of mosquitoes at same specific site
- User Acceptability
  - Suitable for routine use
  - Portable and executable at field level
  - Results obtained on site on less than 5 minutes
  - Minimal training required
  - Low upfront costs
- Safety
  - Safe for use in or near Human dwellings
- Target price
  - Reduces human and budget resources to Plasmodium detection and identification in adult Anopheles spp
  - Reduce labor and resources below 20% of current Plasmodium detection and identification

**DRAFT TPP for non-bioassay technique to quantify surface active ingredients.**

- Goal: Develop novel Develop novel alternative quantitative non-bioassay technique to determine the quantity of active ingredient on a surface available to mosquitoes
  - Cost effective alternative to WHO cone bioassays and current chemical extraction and analysis techniques. Results to drive optimized quality control and operational decisions
- Application method:
  - TBD; Conducted both indoor and outdoor
- Expected performance:
  - Results obtained on site on less than 5 minutes
  - Compatible with detection of all WHO/USEPA recommended insecticides for use in vector control
  - Able to distinguish stereoisomers and quantify concentration of each active ingredient
  - Not interfered by contaminates e.g. dirt or smoke
  - Does not cause damage to solid surfaces like walls, LLINs, etc.
  - Detect all active ingredients on all surfaces
  - Data digitally recorded on site
  - Results consistent with 99% precision in identifying specific compounds. Able to distinguish degradation products and non-active molecules
- User Acceptability
  - Suitable for routine use
  - Upfront costs not a barrier for purchase and use
  - Stable and consistent performance under typical operating conditions in malaria endemic countries
  - Automation desired, e.g. data collected and stored electronically
  - Minimal training required
  - Portable and executable at field level
- Safety
  - Collection device safe for use in or near Human dwellings
- Target price
  - Reduces human and budget resources below current costs to determine surface insecticide
  - Non -bioassays methods may offer advantages of not requiring large costs of maintaining laboratory colonies of mosquitoes and transport of mosquitoes to field locations

**DRAFT TPP to determine insecticide resistance in adult mosquitoes**

- Goal: Develop novel alternative for determining insecticide resistance in adult mosquitoes
  - Cost effective alternative to insecticide resistance testing that reduces human and budget resources over current WHOP tube and CDC bottle assays plus detection of the mechanism by molecular or biochemical tests for molecular markers
- Application method:
  - Conducted both in field and/or in laboratory
- Expected performance:
  - Determine presence of resistance
  - Determine resistance frequency
  - Determine resistance intensity
  - Determine and define resistance mechanism or mechanisms
- User Acceptability
  - Suitable for routine use
  - No need to separate morphologically prior to testing
  - Stable and consistent performance under typical operating conditions in malaria endemic countries
  - Automation desired, e.g. data collected and stored electronically
  - Minimal training required
- Safety
  - Safe for use in or near human dwellings
- Target price
  - Reduces human and budget resources below current costs to determine insecticide resistance
  - Equivalent or less than CDC and WHO bottle assays plus PCR
